# Changes in the mode of travel to work and the severity of depressive symptoms: a longitudinal analysis of UK Biobank

**DOI:** 10.1016/j.ypmed.2018.03.018

**Published:** 2018-07

**Authors:** Craig S. Knott, Jenna Panter, Louise Foley, David Ogilvie

**Affiliations:** MRC Epidemiology Unit and Centre for Diet and Activity Research (CEDAR), University of Cambridge School of Clinical Medicine, Cambridge Biomedical Campus, CB2 0QQ, United Kingdom

**Keywords:** Physical activity, Active commuting, Active travel, Walking, Cycling, Depression, Depression severity, Mental health

## Abstract

Although commuting provides an opportunity for incorporating physical activity into daily routines, little is known about the effect of active commuting upon depressive symptoms. This study aimed to determine whether changes in commute mode are associated with differences in the severity of depressive symptoms in working adults.

Commuters were selected from the UK Biobank cohort if they completed ≥2 assessment centre visits between 2006 and 2016.

Modes of travel to work were self-reported at each visit. Participants were categorised as ‘inactive’ (car only) or ‘active’ commuters (any other mode(s), including walking, cycling and public transport). Transitions between categories were defined between pairs of visits.

The severity of depressive symptoms was defined using the two-item Patient Health Questionnaire (PHQ-2). Scores were derived between zero and six. Higher values indicate more severe symptoms. Separate analyses were conducted in commuters who were asymptomatic (zero score) and symptomatic (non-zero score) at baseline.

The analytical sample comprised 5474 participants aged 40–75 at baseline with a mean follow-up of 4.65 years. Asymptomatic commuters who transitioned from inactive to active commuting reported less severe symptoms at follow-up than those who remained inactive (β −0.10, 95% CI [−0.20, 0.00]; N = 3145). A similar but non-significant relationship is evident among commuters with pre-existing symptoms (β −0.60, 95% CI [−1.27, 0.08]; N = 1078). After adjusting for transition category, longer commutes at baseline were associated with worse depressive symptoms at follow-up among symptomatic participants.

Shifting from exclusive car use towards more active commuting may help prevent and attenuate depressive symptoms in working adults.

## Highlights

• Research shows favourable associations between physical activity and depression.
• Active commuting represents an opportunity for increasing physical activity.
• Shifts to more active modes of travel may attenuate depressive symptoms.
• This is consistent with wellbeing studies and physical activity trials.

## 1 Introduction

With an estimated 298 million cases in 2010 (Ferrari et al., 2013a), depression represents the second leading global cause of years lived with disability (YLD) – a burden that is greatest among those of working age (Ferrari et al., 2013b). Alongside pharmacotherapy and psychotherapy (Karyotaki et al., 2016; Khan et al., 2012), exercise is now recommended as an adjunctive therapy for mild-to-moderate depression (Cleare et al., 2015), with the latest Cochrane review of exercise and depression reporting a moderate effect in favour of exercise (Cooney et al., 2013). Subsequent meta-analyses report larger favourable effects (Honey, 2015; Schuch et al., 2016), while evidence from prospective observational studies suggest that physical activity may also help prevent the development of depressive symptoms (Mammen and Faulkner, 2013).

Efforts to promote physical activity have tended to focus upon active leisure pursuits (Dora and Phillips, 2000), but access, cost and time constraints serve as barriers to uptake (Anokye et al., 2014; Brown and Roberts, 2011). As physical activity is more likely to be sustained when incorporated into everyday routines (Hillsdon and Thorogood, 1996), active commuting has attracted attention in public health strategies (Global Advocacy Council for Physical Activity International Society for Physical Activity and Health, 2010; Public Health England, 2014). Though intuitive, a protective relationship between active commuting and the severity of depressive symptoms should not be assumed given the myriad stressors that may be experienced while walking, cycling and using public transport, including crowding, pollution and poor weather (Evans et al., 2002; Gatersleben and Uzzell, 2007; Koslowsky et al., 1995; Lyons and Chatterjee, 2008; Wener et al., 2003; Rüger et al., 2017).

Existing studies have focussed upon the relationship between commuting and various measures of psychological wellbeing and quality-of-life, capturing domains including life satisfaction, anxiety and social dysfunction. Where reported cross-sectionally, these relationships are inconsistent (Gómez et al., 2013; Humphreys et al., 2013; Office for National Statistics, 2014), while prospective studies show positive associations for cycling versus not cycling (Mytton et al., 2016) and walking or public transport use versus car use (Martin et al., 2014), especially over longer distances (Mytton et al., 2016; Martin et al., 2014). Importantly, only one prospective study has examined changes in commute mode (Martin et al., 2014). Here, commuters who switched from car use to walking reported higher subjective wellbeing than those who maintained their car use. These observational results are supported by a randomized controlled trial of a walking intervention for commuters based on a self-help brochure (Mutrie et al., 2002). Elsewhere, several analyses indicate that both physical activity and metabolic energy expenditure are greater when using alternatives to the car, such as public transport, rather than taking car-only trips to work (Rissel et al., 2012; Langlois et al., 2016; Costa et al., 2015).

However, research that pertains specifically to the severity of depressive symptoms is lacking. We therefore build upon studies of more general measures of psychological wellbeing (Mytton et al., 2016; Martin et al., 2014) by reporting differences in the severity of depressive symptoms between groups of commuters who changed or did not change their mode of travel over time. Analyses are reported separately for commuters who were asymptomatic and symptomatic at baseline, allowing a comparison of the contribution of shifts to more active commutes upon both the development and progression of depressive symptoms. Moderating influences of commute distance and frequency are also reported. Based upon the existing literature for physical activity and commuting, it was hypothesised that the severity of depressive symptoms would be lower at follow-up among participants who transitioned from exclusive car use to a more active mode of travel, particularly among those with longer commutes.

## 2 Methods

### 2.1 Study population

UK Biobank is a population-based prospective cohort of adults aged 37–73 years at recruitment. Participants were invited if they were registered with the National Health Service and lived ≤35 km from one of 22 assessment centres. Of those invited, 502,633 (5.5%) attended an assessment centre between March 2006 and October 2010 to complete a questionnaire concerning their demographic and lifestyle characteristics, medical history and self-rated health. Study design and sampling are detailed elsewhere (Allen et al., 2012; Biobank, 2007).

Participants living ≤35 km from the Stockport assessment centre in the north of England were invited to two repeat assessments, one between December 2009 and June 2013 (n = 20,346) and the other between April 2014 and November 2016 (n = 11,923) (Biobank, 2013).

### 2.2 Exposure

Participants who reported being self-employed or in paid employment were asked at each assessment about the frequency of trips from home to work (trips/week), the distance travelled (miles), and the mode(s) of transport used (‘car or motor vehicle’ (hereafter ‘car’, for simplicity), ‘public transport’, ‘walk’ and/or ‘cycle’).

Modes of travel were first dichotomised as ‘inactive’ (car only) or ‘active’ (any other mode or combination of modes), with each pair of consecutive observations then assigned to one of four groups: (i) consistent travel by car only (hereafter ‘stable inactive’, for simplicity); (ii) consistent use of any other mode or combination of modes (‘stable active’); (iii) switch from exclusive use of a car to any other pattern (‘inactive to active’); or (iv) switch to the exclusive use of a car (‘active to inactive’).

Commuters who participated at all three time points thus provide information for two periods during which a transition could occur (hereafter referred to as 'transition periods'). For any given transition period, the term ‘baseline’ hereafter refers to the first phase of observation and ‘follow-up’ to second phase of observation. Adults who commuted less than once a week or ‘zero’ miles were excluded as home workers.

### 2.3 Outcome

The severity of depressive symptoms was operationalised using the two-item Patient Health Questionnaire (PHQ-2), which has been validated for the assessment of depressive symptom severity and change in symptom severity (Kroenke et al., 2003; Kroenke et al., 2010; Mitchell et al., 2016; Löwe et al., 2005). The instrument asks participants how often they ‘felt down, depressed or hopeless’ or ‘had little interest or pleasure in doing things’ during the preceding two weeks. Response options are: 0 ‘not at all’, 1 ‘several days’, 2 ‘more than half the days’, and 3 ‘nearly every day’. Scores are summed to derive a value between zero and six, with a higher number indicating more severe symptoms (Kroenke et al., 2003). Symptomatic participants were defined as those who reported any symptoms at baseline (i.e. a score > 0).

### 2.4 Covariates

Three groups of variables were included: (i) socio-demographic and occupational characteristics (age-squared, education, ethnicity, household income, marital status, occupational grade, sex and working hours); (ii) lifestyle factors (alcohol consumption, body mass index, non-commuting mode(s) of transport, smoking status, vigorous physical activity, walking for pleasure and workplace physical activity); and (iii) health conditions (bone fracture and ever having been diagnosed with (a) a vascular or (b) a non-vascular health complaint). Age-squared was selected owing to the inverse U-shaped relationship between age and major depressive disorder (Ferrari et al., 2013b). Consistent with diagnostic criteria for depression (American Psychiatric Association, 2013), adjustment was also made for: ‘serious illness, injury or assault’ to the self or a close relative; a death of a close relative, spouse or partner; or financial difficulty in the preceding two years. A continuous variable was also included that accounts for differences in the time elapsed between pairs of observations, denoted by the period of time between two consecutive phases of observation and defined according to the dates of assessment. All other covariates were defined using values reported at the baseline of each pair of observations (Appendix 1).

### 2.5 Statistical analysis

To determine associations between changes in travel mode and the development or progression of depressive symptoms, models were estimated separately for asymptomatic and symptomatic participants. The -xtset- command was used in Stata 14 to declare that the data were longitudinal with repeated observations clustered within individuals (StataCorp, 2015). The within-person relationship between changes in mode of travel and differences in depressive symptomatology were then estimated using a series of linear fixed effects models via the -xtreg- package (StataCorp, 2015). Though similar to linear regression, the fixed effects approach has the added benefit of overcoming the potential issue of differences in depression severity being attributable to unobserved time-invariant differences present between individuals.

Relative differences in depressive symptoms at follow-up were estimated by comparing: (i) participants reporting a transition from ‘inactive to active’ with those in the ‘stable inactive’ group; (ii) those reporting an ‘active to inactive’ transition with those in the ‘stable active’ group; and (iii) those in the ‘stable active’ group with those in the ‘stable inactive’ group.

Covariates were added incrementally: Model 1 (baseline severity of depressive symptoms); Model 2 (as Model 1, plus baseline commute distance and frequency); Model 3 (as Model 2, plus age-squared and time to follow-up); Model 4 (as Model 3, plus socio-demographic, occupational, lifestyle and health-related covariates). All were constrained to the sample of the maximally-adjusted model.

To explore whether the associations differed according to the distance or frequency of travel, interactions were included between changes in travel mode and either baseline commute distance or commute frequency. Robust standard errors are reported.

## 3 Results

### 3.1 Descriptive analysis

**Fig. 1:**
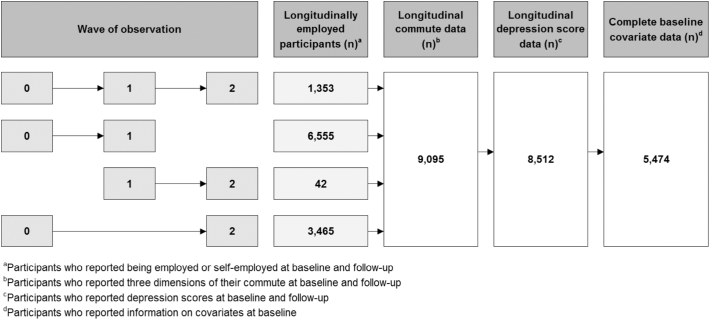
Derivation of the analytical UK Biobank sample.

In total, 11,415 of the 502,633 baseline participants attended at least one repeat assessment and remained in employment (Fig. 1). The analytical sample comprised 5474 participants, capturing 5855 transition periods with a mean follow-up interval of 4.65 years (SD 1.55).

Commuters within the analytical sample were aged between 40 and 75 years in their first phase of complete case participation. Relative to those who used more active modes of travel at baseline, employees who commuted exclusively by car reported higher adiposity and a lower duration of both vigorous physical activity and walking for pleasure at baseline, regardless of whether or not they were defined as symptomatic (Table 1). Employees who were more likely to undertake leisure-time physical activity thus also appeared more likely to commute actively. Consistent results are reported in Appendices 2a and 2b, which present baseline characteristics for each of the four transition categories.

**Table 1:** Descriptive characteristics of the analytical UK Biobank sample at baseline (n = 5474, UK Biobank 2006 to 2016).

	Asymptomatic at baseline			Symptomatic at baseline		
	Active^a^ (N = 1224)	Inactive^a^ (N = 3145)	Difference	Active^a^ (N = 408)	Inactive^a^ (N = 1078)	Difference
Baseline covariates	% (95% CI)	% (95% CI)	p-value	% (95% CI)	% (95% CI)	p-value
*Commute characteristics*						
Commute distance						
Mean (miles)	10.66 (9.21, 12.11)	15.30 (14.12, 16.48)	<0.001	10.52 (7.82, 13.22)	12.28 (11.34, 13.22)	0.227
Commute frequency						
Mean (trips/week)	4.58 (4.50, 4.66)	4.60 (4.55, 4.66)	0.613	4.53 (4.43, 4.64)	4.66 (4.57, 4.74)	0.077

*Severity of depressive symptoms*						
Depression score						
Mean	–	–		1.69 (1.60, 1.79)	1.82 (1.76, 1.88)	0.026

*Socio-demographic and occupational factors*						
Age						
Mean (years)	50.69 (50.32, 51.05)	50.94 (50.70, 51.17)	0.252	49.48 (48.93, 50.04)	50.17 (49.80, 50.54)	0.040
Ethnicity						
White	97.30 (96.16, 98.12)	97.27 (96.59, 97.81)	0.946	95.34 (92.80, 97.02)	95.83 (94.45, 96.87)	0.690
Non-white	2.70 (1.88, 3.84)	2.73 (2.19, 3.41)		4.66 (2.98, 7.20)	4.17 (3.13, 5.55)	
Gross household income						
<£18,000	3.76 (2.82, 4.99)	2.80 (2.27, 3.44)	0.261	6.13 (4.16, 8.93)	3.25 (2.34, 4.49)	0.163
£18,000–30,999	13.07 (11.24, 15.15)	11.89 (10.77, 13.11)		19.12 (15.54, 23.29)	18.46 (16.21, 20.95)	
£31,000–51,999	30.64 (28.04, 33.37)	30.87 (29.24, 32.56)		34.56 (29.99, 39.43)	33.77 (30.99, 36.66)	
£52,000–100,000	41.91 (39.07, 44.81)	42.13 (40.36, 43.92)		33.58 (29.03, 38.45)	38.13 (35.20, 41.14)	
>£100,000	10.62 (8.92, 12.60)	12.31 (11.13, 13.59)		6.62 (4.57, 9.49)	6.40 (5.05, 8.08)	
Highest educational qualification						
University or college degree	59.72 (56.81, 62.57)	48.81 (46.96, 50.66)	<0.001	57.11 (52.10, 61.97)	43.60 (40.58, 46.67)	<0.001
Further education	14.46 (12.55, 16.61)	13.70 (12.48, 15.02)		13.24 (10.26, 16.91)	15.40 (13.32, 17.73)	
Higher secondary education	14.79 (12.85, 16.96)	19.55 (18.15, 21.04)		15.20 (12.01, 19.04)	20.78 (18.40, 23.38)	
Secondary education	3.68 (2.72, 4.95)	5.21 (4.47, 6.07)		4.66 (2.92, 7.35)	8.16 (6.61, 10.04)	
Vocational qualifications	3.43 (2.54, 4.61)	5.82 (5.03, 6.72)		3.92 (2.41, 6.31)	6.22 (4.90, 7.85)	
Other professional qualifications	2.61 (1.83, 3.71)	3.88 (3.24, 4.64)		2.70 (1.49, 4.82)	3.15 (2.26, 4.38)	
None of the above	1.31 (0.80, 2.13)	3.02 (2.46, 3.70)		3.19 (1.86, 5.42)	2.69 (1.83, 3.94)	
Marital status						
Married or partnered	91.83 (90.12, 93.27)	91.76 (90.71, 92.71)	0.945	87.50 (83.90, 90.39)	86.46 (84.16, 88.46)	0.593
Not married or partnered	8.17 (6.73, 9.88)	8.24 (7.29, 9.29)		12.50 (9.61, 16.10)	13.54 (11.54, 15.84)	
Occupational grade						
Managerial	17.24 (15.16, 19.54)	22.99 (21.49, 24.56)	<0.001	14.71 (11.53, 18.57)	21.43 (19.06, 24.00)	0.032
Professional	34.23 (31.47, 37.10)	29.95 (28.30, 31.66)		28.68 (24.37, 33.41)	29.31 (26.61, 32.17)	
Associate professional	20.18 (17.94, 22.63)	19.27 (17.87, 20.74)		16.42 (13.07, 20.43)	16.79 (14.67, 19.14)	
Administrative and secretarial	15.20 (13.22, 17.41)	12.46 (11.32, 13.71)		21.32 (17.49, 25.73)	14.66 (12.61, 16.98)	
Skilled trades	1.80 (1.16, 2.77)	5.34 (4.58, 6.23)		5.15 (3.37, 7.78)	5.29 (4.10, 6.80)	
Professional services	5.31 (4.13, 6.81)	3.78 (3.16, 4.53)		5.88 (3.97, 8.64)	4.73 (3.59, 6.20)	
Sales and customer service	2.21 (1.52, 3.20)	1.40 (1.04, 1.89)		2.21 (1.15, 4.20)	1.58 (0.96, 2.59)	
Plant and machine operatives	1.55 (0.97, 2.48)	2.93 (2.37, 3.61)		2.70 (1.42, 5.06)	3.80 (2.79, 5.16)	
Elementary trades and labourers	2.29 (1.56, 3.34)	1.88 (1.44, 2.43)		2.94 (1.67, 5.12)	2.41 (1.60, 3.62)	
Sex						
Male	53.92 (50.95, 56.86)	52.66 (50.80, 54.50)	0.473	45.59 (40.65, 50.61)	47.59 (44.51, 50.68)	0.501
Female	46.08 (43.14, 49.05)	47.34 (45.50, 49.20)		54.41 (49.39, 59.35)	52.41 (49.32, 55.49)	
Working hours						
Mean (hours/week)	36.13 (25.48, 36.78)	37.34 (36.92, 37.75)	0.002	35.62 (34.65, 36.59)	37.42 (36.72, 38.12)	0.003

*Lifestyle factors*						
Alcohol consumption status						
Current drinker	95.92 (94.54, 96.96)	95.87 (95.08, 96.53)	0.945	93.14 (90.22, 95.23)	95.18 (93.72, 96.31)	0.150
Non-drinker	4.08 (3.04, 5.46)	4.13 (3.47, 4.92)		6.86 (4.77, 9.78)	4.82 (3.69, 6.28)	
Body mass index						
Mean (kg/m^2^)	25.87 (25.64, 26.10)	26.80 (26.63, 26.96)	<0.001	26.15 (25.73, 26.56)	27.19 (26.90, 27.49)	<0.001
Heavy manual/physical work						
Always/usually	4.74 (3.68, 6.08)	7.85 (6.94, 8.87)	<0.001	8.82 (6.38, 12.09)	9.37 (7.70, 11.36)	0.750
Sometimes/rarely/never	95.26 (93.92, 96.32)	92.15 (91.13, 93.06)		91.18 (87.91, 93.62)	90.63 (88.64, 92.30)	
Mainly walking or standing at work						
Always/usually	20.26 (18.01, 22.71)	25.18 (23.64, 26.79)	<0.001	25.98 (21.88, 30.55)	27.83 (25.16, 30.67)	0.479
Sometimes/rarely/never	79.74 (77.29, 81.99)	74.82 (73.21, 76.36)		74.02 (69.45, 78.12)	72.17 (69.33, 74.84)	
Non-commuting modes of transport						
Active	74.59 (72.04, 76.98)	40.25 (38.53, 42.01)	<0.001	74.85 (70.32, 78.73)	39.24 (36.32, 42.23)	<0.001
Inactive	25.41 (23.02, 27.96)	59.75 (57.99, 61.47)		25.25 (21.27, 29.68)	60.76 (57.76, 63.68)	
Smoking status						
Current smoker	5.56 (4.37, 7.04)	5.82 (5.04, 6.71)	0.739	8.82 (6.32, 12.20)	7.98 (6.47, 9.80)	0.615
Non-smoker	94.44 (92.96, 95.63)	94.18 (93.29, 94.96)		91.18 (87.80, 93.68)	92.02 (90.20, 93.53)	
Vigorous physical activity						
Mean (minutes/week)	33.22 (31.10, 35.33)	27.53 (26.26, 28.81)	<0.001	28.00 (24.67, 31.32)	23.71 (21.60, 25.82)	0.032
Walking for pleasure						
Median (minutes/week)	28.13 (0.00, 101.25)^e^	26.25 (0.00, 101.25)^e^	0.072	28.13 (0.00, 101.25)^e^	18.75 (0.00, 75.00)^e^	0.005

*Health factors*						
Bereavement in the preceding two years						
Yes	19.36 (17.25, 21.67)	20.70 (19.32, 22.15)	0.317	21.08 (17.35, 25.37)	22.36 (19.97, 24.94)	0.595
No	80.64 (78.33, 82.75)	79.30 (77.85, 80.68)		78.92 (74.63, 82.65)	77.64 (75.06, 80.03)	
Bone fracture in the preceding five years						
Yes	7.03 (5.68, 8.66)	7.38 (6.50, 8.36)	0.694	9.80 (7.27, 13.09)	5.84 (4.59, 7.42)	0.016
No	92.97 (91.34, 94.32)	92.62 (91.64, 93.50)		90.20 (86.91, 92.73)	94.16 (92.58, 95.41)	
Financial difficulty in the preceding two years						
Yes	7.68 (6.30, 9.33)	8.59 (7.64, 9.63)	0.326	20.83 (17.15, 25.07)	21.24 (18.87, 23.83)	0.862
No	92.32 (90.67, 93.70)	91.41 (90.37, 92.36)		79.17 (74.93, 82.85)	78.76 (76.17, 81.13)	
Non-vascular condition or disability^b^						
Yes	20.59 (16.90, 24.84)	17.55 (16.21, 18.98)	0.859	20.59 (16.90, 24.84)	21.34 (18.95, 23.93)	0.753
No	79.41 (75.16, 83.10)	82.45 (81.02, 83.79)		79.41 (75.16, 83.10)	78.66 (76.07, 81.05)	
Serious illness or injury in the preceding two years^c^						
Yes	20.51 (18.30, 22.90)	17.65 (16.35, 19.03)	0.035	24.51 (20.53, 28.99)	21.34 (18.97, 23.91)	0.203
No	79.49 (77.10, 81.70)	82.35 (80.97, 83.65)		75.49 (71.01, 79.47)	78.66 (76.09, 81.03)	
Vascular condition^d^						
Yes	13.32 (11.47, 15.40)	16.85 (15.53, 18.26)	0.004	17.16 (13.70, 21.27)	16.70 (14.51, 19.14)	0.839
No	86.68 (84.60, 88.53)	83.15 (81.74, 84.47)		82.84 (78.73, 86.30)	83.30 (80.86, 85.49)	

N refers to the total number of observed transition periods. Differences in covariate means and proportions by baseline commute mode were tested by way of a Wald test.

a Inactive commuting defined as any commute by ‘car or motor vehicle’ only, with active commuting defined as using any other mode or combination of modes.

b Defined according to whether participants reported ever receiving a doctor's diagnosis for diabetes, cancer or ‘any other serious medical conditions or disabilities’.

c Defined as any self-reported ‘serious illness or injury’ to the participant or a close relative in the two years preceding baseline.

d Defined according to whether participants reported ever receiving a doctor's diagnosis for angina, heart attack, high blood pressure or stroke.

e Median and inter-quartile range reported, with differences in distributions assessed using the Wilcoxon rank-sum test.

Mean depression score increased to 0.22 (95% CI [0.20, 0.24]) by the end of follow-up among participants who were asymptomatic at baseline. For those who were symptomatic at baseline, scores fell from 1.78 (95% CI [1.73, 1.84]) to 1.00 (95% CI [0.93, 1.07]). Within both groups, mean changes in depressive symptom scores were broadly similar across all transition categories (Appendix 3).

Regardless of baseline symptomatology, commuting exclusively by car was the most common form of travel (asymptomatic: 64.1%; symptomatic: 63.2%). Cycling only or walking and cycling were the least prevalent commute modes (asymptomatic: 0.2%; symptomatic: 0.1%). Most participants (84.7%) reported no change in travel mode between time points (Appendix 4).

### 3.2 Associations between commuting transitions and severity of depressive symptoms

Table 2 reports associations between changes to the mode of travel and relative differences in the severity of depressive symptoms at follow-up. Among participants who were asymptomatic at baseline, those who switched to more active modes of commuting tended to report a lower severity of symptoms at follow-up than those who continued to travel inactively (β −0.10, 95% CI [−0.20, 0.00]; Table 2). A similar relationship was evident among commuters with pre-existing symptoms (β −0.60, 95% CI [−1.27, 0.08]), though this was not statistically significant. In neither group was a transition in the opposite direction associated with a reverse effect on symptoms.

**Table 2:** Associations between depressive symptoms at follow-up and changes in travel mode in commuters with and without symptoms at baseline (n = 5474, UK Biobank 2006 to 2016).

		Model 1^b^		Model 2^c^		Model 3^d^		Model 4^e^	
	Sample (N)	β (95% CI)	p-value	β (95% CI)	p-value	β (95% CI)	p-value	β (95% CI)	p-value
*Asymptomatic at baseline*									
Inactive at baseline^a^									
Stable inactive	2800	Reference		Reference		Reference		Reference	
Inactive to active	345	−0.06 (−0.14, 0.02)	0.172	−0.05 (−0.13, 0.02)	0.180	−0.05 (−0.13, 0.03)	0.216	−0.10 (−0.20, 0.00)	0.056
Active at baseline^a^									
Stable active	924	Reference		Reference		Reference		Reference	
Active to inactive	300	−0.11 (−0.34, 0.13)	0.372	−0.10 (−0.34, 0.14)	0.418	−0.07 (−0.29, 0.15)	0.513	−0.05 (−0.28, 0.17)	0.634
No transition									
Stable inactive	2800	Reference		Reference		Reference		Reference	
Stable active	924	0.04 (−0.20, 0.29)	0.743	0.04 (−0.21, 0.28)	0.775	0.00 (−0.24, 0.24)	0.975	−0.03 (−0.26, 0.20)	0.784
Commute distance									
Difference per 10-mile increase in commute distance	4369	–		0.01 (−0.01, 0.02)	0.270	0.00 (−0.01, 0.02)	0.552	0.00 (−0.01, 0.01)	0.872
Commute frequency									
Difference per additional trip to work per week	4369	–		−0.01 (−0.03, 0.01)	0.471	0.01 (−0.01, 0.03)	0.282	0.01 (−0.02, 0.04)	0.641

*Symptomatic at baseline*									
Inactive at baseline^a^									
Stable inactive	939	Reference		Reference		Reference		Reference	
Inactive to active	139	−0.93 (−1.68, −0.17)	0.016	−0.90 (−1.65, −0.15)	0.018	−0.74 (−1.38, −0.11)	0.021	−0.60 (−1.27, 0.08)	0.082
Active at baseline^a^									
Stable active	296	Reference		Reference		Reference		Reference	
Active to inactive	112	−0.07 (−1.33, 1.19)	0.914	−0.03 (−1.19, 1.13)	0.961	0.21 (−0.65, 1.07)	0.630	−0.19 (−1.25, 0.87)	0.728
No transition									
Stable inactive	939	Reference		Reference		Reference		Reference	
Stable active	296	−0.12 (−1.65, 1.42)	0.883	−0.18 (−1.65, 1.29)	0.812	−0.61 (−1.72, 0.49)	0.277	−0.15 (−1.52, 1.22)	0.831
Commute distance									
Difference per 10-mile increase in commute distance	1486	–		−0.06 (−0.51, 0.39)	0.786	−0.10 (−0.44, 0.25)	0.589	0.64 (0.13, 1.16)	0.014
Commute frequency									
Difference per additional trip to work per week	1486	–		−0.12 (−0.46, 0.22)	0.489	−0.19 (−0.49, 0.10)	0.193	0.04 (−0.36, 0.43)	0.862

N refers to the total number of observed transition periods.

a Inactive commuting defined as any commute by ‘car or motor vehicle’ only, with active commuting defined as using any other mode or combination of modes.

b Adjusted for baseline depression score.

c As Model 1, plus baseline commute distance and commute frequency.

d As Model 2, plus baseline age-squared and time to follow-up. Age-squared was selected owing to the inverse U-shaped relationship between age and major depressive disorder (Ferrari et al., 2013b).

e As Model 3, plus baseline socio-demographic and occupational factors (education, hours worked per week, marital status, occupational grade), lifestyle factors (alcohol consumption status, body mass index, heavy or manual physical activity at work, mode of non-commuting transport, smoking status, walking or standing at work, weekly duration of vigorous physical activity, weekly duration of walking for pleasure) and health status factors (bereavement in the two years preceding baseline, bone fracture in the five years preceding baseline, ever-diagnosis of a non-vascular condition (diabetes, cancer or ‘any other serious medical conditions or disabilities’), ever-diagnosis of a vascular condition (angina, heart attack, high blood pressure or stroke), financial difficulty in the two years preceding baseline, self-reported ‘serious illness or injury’ to the participant or a close relative in the two years preceding baseline).

Among commuters who were symptomatic at baseline, longer journeys at baseline were associated with worse symptoms at follow-up (β 0.64 for each additional 10 miles, 95% CI [0.13, 1.16]). No such association was observed in asymptomatic participants, and commuting frequency was not associated with depressive symptomatology in either group.

### 3.3 Interactions with distance and frequency of commuting

As reported in Table 3, no interaction was found between distance and changes in travel mode among commuters without pre-existing symptoms, but higher commuting frequencies at baseline appear to be associated with less severe symptoms at follow-up among those making a transition from inactive to active commuting (β −0.12 for each additional trip per week, 95% CI [−0.26, 0.01]).

**Table 3:** Interactions between changes in travel mode and the distance and frequency of travel at baseline in commuters with and without symptoms at baseline (n = 5474, UK Biobank 2006 to 2016.)

		Model 1^b^		Model 2^c^	
Commute transitions by baseline symptomatology	Sample (N)	Coefficient (95% CI)	p-value	Coefficient (95% CI)	p-value
Asymptomatic at baseline					
Inactive at baseline^a^					
Inactive to active, relative to stable inactive	3145				
*Difference per 10-mile increase in baseline commute distance*		0.02 (−0.02, 0.07)	0.252	−0.02 (−0.10, 0.05)	0.573
*Difference per one trip increase in weekly baseline commute frequency*		−0.10 (−0.21, 0.01)	0.085	−0.12 (−0.26, 0.01)	0.071
Active at baseline^a^					
Active to inactive, relative to stable active	1224				
*Difference per 10-mile increase in baseline commute distance*		0.03 (−0.02, 0.08)	0.250	0.03 (−0.03, 0.10)	0.268
*Difference per one trip increase in weekly baseline commute frequency*		0.04 (−0.03, 0.11)	0.233	0.01 (−0.06, 0.08)	0.777
No transition					
Stable active, relative to stable inactive	3724				
*Difference per 10-mile increase in baseline commute distance*		0.00 (−0.01, 0.01)	0.841	0.00 (−0.02, 0.01)	0.843
*Difference per one trip increase in weekly baseline commute frequency*		0.00 (−0.01, 0.02)	0.381	0.00 (−0.03, 0.04)	0.853

*Symptomatic at baseline*					
Inactive at baseline^a^					
Inactive to active, relative to stable inactive	1078				
*Difference per 10-mile increase in baseline commute distance*		−0.87 (−1.53, −0.21)	0.010	0.27 (−0.93, 1.47)	0.661
*Difference per one trip increase in weekly baseline commute frequency*		−0.51 (−1.00, −0.02)	0.040	0.33 (−0.84, 1.49)	0.582
Active at baseline^a^					
Active to inactive, relative to stable active	408				
*Difference per 10-mile increase in baseline commute distance*		0.10 (−0.45, 0.65)	0.721	0.48 (−0.02, 0.98)	0.059
*Difference per one trip increase in weekly baseline commute frequency*		−0.28 (−0.84, 0.27)	0.319	0.55 (−0.57, 1.66)	0.335
No transition					
Stable active, relative to stable inactive	1235				
*Difference per 10-mile increase in baseline commute distance*		0.67 (0.18, 1.16)	0.008	1.35 (0.32, 2.38)	0.010
*Difference per one trip increase in weekly baseline commute frequency*		0.10 (−0.86, 1.07)	0.834	0.61 (−0.48, 1.71)	0.272

N refers to the total number of observed transition periods.

a Inactive commuting defined as any commute by ‘car or motor vehicle’ only, with active commuting defined as using any other mode or combination of modes.

b Model includes adjustment for baseline depression score and an interaction between modal transition and either baseline commute frequency or distance.

c As Model 1, plus adjustment for baseline age-squared and time to follow-up, baseline demographic and occupational factors (education, hours worked per week, marital status, occupational grade), baseline lifestyle factors (alcohol consumption status, body mass index, heavy or manual physical activity at work, mode of non-commuting transport, smoking status, walking or standing at work, weekly duration of vigorous physical activity, weekly duration of walking for pleasure), and baseline health status (bereavement in the two years preceding baseline, bone fracture in the five years preceding baseline, ever-diagnosis of a non-vascular condition (diabetes, cancer or “any other serious medical conditions or disabilities”), ever-diagnosis of a vascular condition (angina, heart attack, high blood pressure or stroke), financial difficulty in the two years preceding baseline, self-reported “serious illness or injury” to the participant or a close relative in the two years preceding baseline).

Of participants with pre-existing symptoms, there is an indication that a transition from active to inactive commuting may be associated with more severe depressive symptoms at follow-up among those who travelled further to work at baseline (β 0.48 for each additional 10 miles, 95% CI [−0.02, 0.98]). In addition, longer journeys at baseline were associated with more severe symptoms at follow-up in ‘stable active’ commuters compared to the ‘stable inactive’ (β 1.35 for each additional 10 miles, 95% CI [0.32, 2.38]).

## 4 Discussion

This study investigated the associations between changes in the mode of travel to work and the severity of depressive symptoms at follow-up in a cohort of adult commuters with and without symptoms at baseline. Following adjustment for socio-demographic, lifestyle and health-related factors, findings are consistent with the hypothesis that a transition from travel exclusively by car to more active forms of commuting may contribute to an attenuation of both the development and the progression of depressive symptoms. Among participants with pre-existing symptoms, for example, switching from inactive to active commuting appears to be associated with a PHQ-2 score that was 0.60 units lower at follow-up than among commuters who remained inactive between phases. Although no guidelines currently exist for determining the clinical significance of differences or changes in PHQ-2 score within asymptomatic and symptomatic general populations, these positive findings are harmonious with results of other longitudinal studies, which suggest that active commuting may benefit subjective wellbeing (Mytton et al., 2016; Martin et al., 2014; Mutrie et al., 2002). They are also in keeping with results from therapeutic trials of physical activity for depression (Cooney et al., 2013; Honey, 2015; Helgadóttir et al., 2017), notwithstanding some dispute as to its efficacy alongside standard care (Daley and Jolly, 2012).

While commuting offers a convenient way of incorporating physical activity into day-to-day life (Hillsdon and Thorogood, 1996), the precise mechanism(s) by which transitions to more active forms of travel may prevent or ameliorate depressive symptoms are unclear. Possible pathways include the effect of increases in physical activity (Foley et al., 2015; Sahlqvist et al., 2012) upon improved physical health and thereby the severity of depressive symptoms (Celis-Morales et al., 2017; Møller et al., 2011), to a reduction in exposure to adverse events associated with commuting by car (Gatersleben and Uzzell, 2007; Lyons and Chatterjee, 2008; Christian, 2012a; Gottholmseder et al., 2009; Mattisson et al., 2015). The validity of the first hypothesis has recently been called into question by a new study of exercise and depression risk, which found that reductions in risk were not explained by differences in a marker for cardiovascular fitness, suggesting that the benefits of exercise may operate through a mechanism other than its physiological impact (Harvey et al., 2017).Additional studies are required to replicate these findings. These and studies of other plausible mediators are an important area for further research. Whatever the mechanism(s), it should be borne in mind that adults with, or at risk of developing, depressive symptoms might experience greater difficulty initiating or sustaining active routines (Blumenthal et al., 2012; Vancampfort et al., 2015) and may therefore benefit from targeted and individualised support (Blumenthal et al., 2012).

Irrespective of travel mode, symptoms appear to be worse at following among symptomatic participants who undertook longer commutes at baseline. This finding is concordant with studies that report less relaxation (Gottholmseder et al., 2009), physical activity, sleep (Christian, 2012b) and social connectivity (Christian, 2012a) among adults who commute further or for longer durations. Among participants with pre-existing symptoms, the effect of distance differed in at least two notable ways between those who switched travel mode and those who did not.

First, of active commuters who travelled longer distances at baseline, those who switched to an inactive mode of travel reported worse symptoms at follow-up than participants who continued to travel actively. Though not statistically significant, this appears concordant with longitudinal research that reports a negative association between time spent driving and mental wellbeing (Martin et al., 2014), possibly resulting from negative experiential consequences of long car journeys. Second, over longer baseline distances, the consistent use of active commuting was associated with worse symptoms than stable inactive travel. Given that active commuting is more likely over shorter distances (Badland et al., 2008; Dalton et al., 2013; Ogilvie et al., 2008; Yang et al., 2015), and less likely in households of greater affluence (Goodman, 2013) and car ownership (Dalton et al., 2013; Ogilvie et al., 2008), some participants who commuted actively over longer distances may have done so out of necessity rather than choice. This hypothesis is indirectly supported by indications that employees of lower socio-economic position may travel further to work owing to the cost of living in more proximate areas (Goodman et al., 2012), suggesting a potential benefit to depressive symptomatology from housing and labour market policies that reduce the requirement for longer commutes. However, within UK Biobank, there was no apparent difference in baseline income or financial difficulty between transition categories (Appendices 2a and 2b), and no relationship between baseline commute distance and household income (asymptomatic: p = 0.434; symptomatic p = 0.366; results not shown). Although these interactions appear to conflict with previous prospective analyses (Mytton et al., 2016; Martin et al., 2014), which favour longer and more active commutes, discrepancies may reflect differences in study populations and the operationalisation of variables. Also of note is the finding that, relative to stable inactive commuters, the severity of depressive symptoms was greater at follow-up at longer distances among stable active commuters. Assuming that this difference in effect was not attributable to between-group confounding, such as markers of socio-economic status, this finding indicates the possibility of a tipping point or optimal threshold for attenuating the severity of depressive symptoms through active travel. The dose-response relationship between active commuting and depressive symptomatology represents an interesting avenue for future research.

### 4.1 Strengths and limitations

To our knowledge, this is the first study to explore longitudinal associations between changes in travel mode and the severity of depressive symptoms in commuters. This focus upon transitions provides a better indication as to the likely impact of efforts to shift travel behaviours at the population level, and a stronger basis for causal inference than cross-sectional studies. The analysis benefits from a validated measure of depressive symptomatology and adjustment for the effect of physical activity outside the commute and physical health – a factor overlooked by some existing studies (Gómez et al., 2013; Humphreys et al., 2013; Martin et al., 2014). Moreover, given the inverse U-shaped association between age and major depressive disorder (Ferrari et al., 2013b), it is likely that participants sampled within the UK Biobank dataset were more likely to depressive symptoms than might be the case in comparable but younger or older cohorts.

Although overall changes in the severity of depressive symptoms are suggestive of regression to the mean (Morton and Torgerson, 2005), adjustment for baseline severity and time to follow-up reduce the likelihood that this could have influenced associations observed in maximally-adjusted models. There is nonetheless a possibility that changes in the severity of depressive symptoms may be in part a consequence of shifts between depression episodes as opposed to any true change in symptoms. Other data-related limitations are also acknowledged. Firstly, as changes in travel mode and depressive symptoms were measured concurrently, reverse causation is possible. However, among asymptomatic participants who reported worsening symptoms over time on average, there was no indication that symptom severity was any different at follow-up among those who transitioned to inactive travel, relative to those who remained inactive (Appendix 3). Unfortunately, owing to the limitations of sample size, we were unable further investigate the reverse causation hypothesis by restricting analyses to symptomatic participants who reported a worsening of symptoms between phases. The pattern of results is nevertheless consistent with the argument that changes to the mode of travel preceded changes to depressive symptoms. Secondly, due to the relatively small number of participants who changed travel mode between pairs of phases, only four transition categories were defined. Although a more detailed range of transition categories were initially considered, a higher level of discrimination between distinct groups of commuters was not achievable with the data available. Likewise, it was not feasible to explore non-linear interactions between commute mode and either the frequency or distance of travel, while other dimensions of the commute (such as commute duration) were unavailable within the UK Biobank cohort. Thirdly, an assumption of fixed effects models is that ‘treatment’ and ‘control’ groups experience the same exposures during follow-up (Dimick and Ryan, 2014; Listl et al., 2016). However, as transport is self-selected, time-varying confounders may be differentially distributed between transition categories, such as changes to income, home address or occupation, which have been associated with differences in markers of psychological wellbeing (Alcock et al., 2014; Boyce and Oswald, 2012; Flint et al., 2013). Being unable to establish the temporal ordering of such changes, adjustment or interaction by time-varying factors was considered inappropriate, particularly given the small number of shifts in such factors documented within each commute mode transition category. As an additional point of note, it should be remembered that measurements at each phase offer snapshots of participant behaviours and characteristics at a specific point in time; it is plausible that these may vary in ways uncaptured by the study during periods between measurements. Fourthly, longitudinal UK Biobank data are geographically limited, and although various environmental characteristics appear to be associated with depression and psychological wellbeing more generally (Galea et al., 2005; Guite et al., 2006; Kim, 2008), adjustment was not undertaken due to lags of up to a decade between measurement and participant enrolment. Though internally valid, results from this study are therefore unlikely to be representative of the UK mid-life population as a whole, and so may not be fully generalisable. Fifthly, asymptomatic and symptomatic groups were not defined in a manner analogous to a positive screen or clinical diagnosis for a condition such as major depressive disorder. Such bifurcation was beyond the purview of this study. Finally, some data were missing. Unfortunately, loss to follow-up could not be established at the time of analysis owing to an absence of data for identifying participants invited for repeat assessments.

## 5 Conclusion

Depression is a common and debilitating mental health condition that affects almost 300 million adults worldwide (Ferrari et al., 2013a), for which physical activity is an important and effective adjunctive therapy (Cleare et al., 2015). The importance of physical activity for the prevention and control of non-communicable disease is recognised both nationally (Public Health England, 2014) and internationally (Global Advocacy Council for Physical Activity International Society for Physical Activity and Health, 2010; United Nations, 2013), with active commuting promoted as a means of decreasing sedentary behaviour (Global Advocacy Council for Physical Activity International Society for Physical Activity and Health, 2010; Public Health England, 2014). However, evidence concerning the efficacy of different interventions for the promotion of active commuting remains inconsistent, with generalisability limited by factors including poor quality, sampling of groups already motivated to change, and the implementation of heterogeneous strategies. Such strategies range from changes to the built environment, such as the development of a new cycle path infrastructure, to workplace-based initiatives that provide on-site changing facilities and subsidised bicycle purchase schemes (Ogilvie et al., 2004; Scheepers et al., 2014; Yang et al., 2010). The targeting of participants amenable to change is likely to be an important limitation in determining the effectiveness of different interventional approaches, given indications within this dataset that employees who are less physically active in their leisure time were also less likely to commute actively. It is possible that interventions may be missing those commuters most in need of behaviour change. Moreover, while the majority of health professionals recognise the importance of promoting physical activity, not all patients receive advice on how to be more active (Nunan, 2016). This apparent disconnect between policy and practice may partly reflect uncertainty among professionals concerning the effectiveness of different behaviour change interventions (Nunan, 2016). This paper marks a forward step in attempting to bridge these gaps in the evidence base, finding that the incorporation of walking, cycling or public transport as part of the commute may contribute to an attenuation of both the development and progression of depressive symptoms in working adults. Future research should investigate the pathways by which active commuting may confer such advantageous effects for depressive symptomatology, and assess the effectiveness of environmental and behavioural interventions. Such evidence will help mental health professionals and transport planners to support adults to take up and maintain active commuting in a manner that is clinically beneficial.

## Contributors

CK, JP, LF and DO were involved in study conception and design. CK completed the data analysis. CK, JP, LF and DO interpreted the data and provided important intellectual input. CK wrote the first draft. CK, JP, LF and DO read, commented on and edited the manuscript. This research has been conducted using the UK Biobank Resource under Application Number 20684.

## Conflicts of interest

None.

## Funding

Craig Knott, Jenna Panter and David Ogilvie were supported by the Medical Research Council (MC_UU_12015/6). LF is supported by the Centre for Diet and Activity Research (CEDAR), a UKCRC Public Health Research Centre of Excellence. Funding from the British Heart Foundation, Cancer Research UK, Economic and Social Research Council, Medical Research Council, the National Institute for Health Research, and the Wellcome Trust, under the auspices of the UK Clinical Research Collaboration, is gratefully acknowledged (087636/Z/08/Z, ES/G007462/1, MR/K023187/1). No funder had any role in the study design; data collection, analysis, or interpretation; in the writing of the report; or in the decision to submit the article for publication.

## Appendix A Supplementary data

Supplementary material Image 1
