# Supplementary material for: Changes in the mode of travel to work and the severity of depressive symptoms: a longitudinal analysis of UK Biobank
Source: Prev Med. 2018 Jul;112:61–9. doi: 10.1016/j.ypmed.2018.03.018 (PMC5999356; doi:10.1016/j.ypmed.2018.03.018)
Supplement: Supplementary file 1 — Supplementary material [file mmc1.docx]

**Appendix 1 Additional methodological information**

Travel modes used to work

Participants were asked: “What types of transport do you use to get to and from work?” They were able to select one or more of the following transport options: car or motor vehicle, walk, public transport, or cycle.

Socio-demographic and occupational factors

Socio-demographic characteristics include age-squared, owing to the inverse U-shaped relationship between age and depressive disorders,^[[1]](#endnote-1)^ gross household income (<£18,000, £18,000-30,999, £31,000-51,999, £52,000-100,000, >£100,000), highest educational qualification (university or college degree, further education [A level or equivalent], higher secondary education [GCSE or equivalent], secondary education [CSEs or equivalent], vocational qualifications [NVQ, Higher National Diploma, Higher National Certificate, or equivalent], other professional qualifications, none of the above) and marital status, which was derived based on the reported relationship of people in the household to the participant (married or partnered, not married or partnered).

The occupational grade of employed participants was defined according to the Standard Occupational Classification 2000^[[2]](#endnote-2)^ (managerial, professional, associate professional and technical, administrative and secretarial, skilled trades, professional services, sales and customer service, plant and machine operators, elementary trades and labourers), with working hours per week captured as integers.

As they were time-invariant within individuals, sex (male, female) and ethnicity (white, non-white) were automatically dropped from all models.

Lifestyle factors

Body mass index (kg/m^2^), smoking status (current smoker, non-smoker) and alcohol consumption status (current drinker, former drinker) were all included as lifestyle factors. In an effort to account for exercise undertaken outside of the commute, leisure- and work-time physical activity variables were also included.

The first leisure-time variable captures the minutes spent walking for pleasure per week, as derived by multiplying the frequency of walking for pleasure for at least 10 minutes per occasion in the last four weeks by the average duration spent walking for pleasure per occasion. In addition to those who reported that they undertook no walking for pleasure in a typical week, participants who stated they were unable to walk were assigned a zero value for their weekly duration walking for pleasure. The second leisure-time variable captures the average duration in minutes of vigorous physical activity during a typical day, defined as ‘activities that make you breathe hard, such as fast cycling, aerobics or heavy lifting’. The daily duration of vigorous physical activity was coded as zero for participants who stated for a preceding question that they undertook less than 10 minutes of vigorous physical activity in a typical week. The final leisure-time variable captures the non-commuting modes of transport used during the four weeks preceding baseline, categorised as ‘active’ or ‘inactive’ using the same method as for the commute. This question was not asked of participants who reported being unable to walk. Such participants were coded as ‘inactive’. Two questions concerning the degree to which participants were involved in ‘heavy manual or physical work’ (always/usually, sometimes/rarely/never) or ‘mainly walking or standing’ (always/usually/sometimes, rarely/never) as part of their employment were used to capture job-related physical activity. Of the physical activity variables available within UK Biobank, these five variables were selected in an effort to limit overlap with physical activity undertaken as part of the commute.

Health status factors

Consistent with the DSM-V diagnostic criteria for MDD, which states that clinical judgement should be used to delineate depressive symptoms from the ‘normal sadness and grief’ experienced as an expectable and proportional response to psychosocial stressors such as financial ruin or bereavement,^[[3]](#endnote-3) ,^^[[4]](#endnote-4)^ three binary variables were created according to whether participants reported (a) a ‘serious illness, injury or assault’ to the self or a close relative, (b) a death of a close relative, spouse or partner, or (c) financial difficulty in the preceding two years.

A binary vascular health variable was also derived, representing whether participants reported ever receiving a diagnosis of angina, a heart attack, high blood pressure or stroke. The effects of non-vascular health conditions were captured via a further binary variable, coded according to whether participants reported ever being diagnosed with diabetes, cancer or ‘any other serious medical conditions or disabilities’. A final binary variable was also created, representing any self-reported bone fracture within the five years preceding baseline.

**Appendix 2a Detailed breakdown of baseline characteristics for each of the four commute transition categories among commuters who were asymptomatic at baseline within the analytical sample**

|  | **Asymptomatic at baseline** | | | |  |
| --- | --- | --- | --- | --- | --- |
|  | **Stable inactive (N=2,800)** | **Stable active (N=924)** | **Inactive to active (N=345)** | **Active to inactive (N=300)** | **Difference** |
| **Baseline covariates** | **% (95% CI)** | **% (95% CI)** | **% (95% CI)** | **% (95% CI)** | **p-value** |
| **Commute characteristics** |  |  |  |  |  |
| **Commute distance** |  |  |  |  |  |
| Mean (miles) | 15.45 (14.16, 16.74) | 9.88 (8.19, 11.56) | 14.11 (11.65, 16.58) | 13.07 (10.20, 15.95) | <0.001 |
|  |  |  |  |  |  |
| **Commute frequency** |  |  |  |  |  |
| Mean (trips/week) | 4.61 (4.56, 4.67) | 4.59 (4.50, 4.69) | 4.54 (4.41, 4.67) | 4.54 (4.39, 4.70) | 0.651 |
|  |  |  |  |  |  |
| **Severity of depressive symptoms** |  |  |  |  |  |
| **Depression score** |  |  |  |  |  |
| Mean | - | - | - | - |  |
|  |  |  |  |  |  |
| **Socio-demographic and occupational factors** |  |  |  |  |  |
| **Age** |  |  |  |  |  |
| Mean (years) | 51.03 (50.79, 51.28) | 50.85 (50.43, 51.28) | 50.14 (49.47, 50.81) | 50.16 (49.48, 50.85) | 0.016 |
|  |  |  |  |  |  |
| **Ethnicity** |  |  |  |  |  |
| White | 97.29 (96.56, 97.86) | 97.51 (96.15, 98.40) | 97.10 (94.68, 98.44) | 96.67 (93.90, 98.20) | 0.914 |
| Non-white | 2.71 (2.14, 3.44) | 2.49 (1.60, 3.85) | 2.90 (1.56, 5.32) | 3.33 (1.80, 6.10) |  |
|  |  |  |  |  |  |
| **Gross household income** |  |  |  |  |  |
| <£18,000 | 2.93 (2.36, 3.63) | 3.90 (2.82, 5.36) | 1.74 (0.78, 3.83) | 3.33 (1.80, 6.10) | 0.323 |
| £18,000-30,999 | 11.86 (10.67, 13.16) | 13.64 (11.50, 16.10) | 12.17 (9.11, 16.09) | 11.33 (8.19, 15.47) |  |
| £31,000-51,999 | 30.86 (29.13, 32.64) | 31.28 (28.25, 34.47) | 31.01 (26.33, 36.12) | 28.67 (23.81, 34.07) |  |
| £52,000-100,000 | 42.43 (40.55, 44.33) | 40.69 (37.43, 44.04) | 39.71 (34.65, 45.00) | 45.67 (40.08, 51.37) |  |
| >£100,000 | 11.93 (10.69, 13.28) | 10.50 (8.54, 12.84) | 15.36 (11.91, 19.59) | 11.00 (7.91, 15.10) |  |
|  |  |  |  |  |  |
| **Highest educational qualification** |  |  |  |  |  |
| University or college degree | 47.75 (45.79, 49.72) | 61.90 (58.54, 65.16) | 57.39 (52.08, 62.53) | 53.00 (47.31, 58.62) | <0.001 |
| Further education | 13.64 (12.35, 15.04) | 14.18 (12.01, 16.66) | 14.20 (10.89, 18.32) | 15.33 (11.66, 19.90) |  |
| Higher secondary education | 20.21 (18.70, 21.82) | 12.66 (10.59, 15.07) | 14.20 (10.89, 18.32) | 21.33 (17.04, 26.36) |  |
| Secondary education | 5.11 (4.33, 6.01) | 3.90 (2.77, 5.45) | 6.09 (3.99, 9.17) | 3.00 (1.56, 5.68) |  |
| Vocational qualifications | 6.29 (5.42, 7.28) | 3.03 (2.10, 4.36) | 2.03 (0.97, 4.21) | 4.67 (2.77, 7.75) |  |
| Other professional qualifications | 3.86 (3.18, 4.67) | 2.92 (2.01, 4.23) | 4.06 (2.41, 6.75) | 1.67 (0.69, 3.96) |  |
| None of the above | 3.14 (2.54, 3.89) | 1.41 (0.82, 2.41) | 2.03 (0.97, 4.21) | 1.00 (0.32, 3.07) |  |
|  |  |  |  |  |  |
| **Marital status** |  |  |  |  |  |
| Married or partnered | 91.93 (90.82, 92.91) | 91.02 (88.93, 92.74) | 90.43 (86.83, 93.13) | 94.33 (91.05, 96.46) | 0.173 |
| Not married or partnered | 8.07 (7.09, 9.18) | 8.98 (7.26, 11.07) | 9.57 (6.87, 13.17) | 5.67 (3.54, 8.95) |  |
|  |  |  |  |  |  |
| **Occupational grade** |  |  |  |  |  |
| Managerial | 23.54 (21.94, 25.21) | 15.04 (12.79, 17.61) | 18.55 (14.78, 23.03) | 24.00 (19.48, 29.19) | <0.001 |
| Professional | 29.29 (27.54, 31.10) | 36.04 (32.80, 39.41) | 35.36 (30.47, 40.58) | 28.67 (23.81, 34.07) |  |
| Associate professional | 19.14 (17.66, 20.71) | 19.37 (16.84, 22.19) | 20.29 (16.36, 24.89) | 22.67 (18.26, 27.78) |  |
| Administrative and secretarial | 12.29 (11.08, 13.60) | 16.13 (13.78, 18.78) | 13.91 (10.63, 18.00) | 12.33 (9.05, 16.59) |  |
| Skilled trades | 5.54 (4.71, 6.50) | 1.30 (0.71, 2.38) | 3.77 (2.19, 6.40) | 3.33 (1.80, 6.10) |  |
| Professional services | 3.89 (3.22, 4.70) | 5.74 (4.34, 7.54) | 2.90 (1.56, 5.32) | 4.00 (2.28, 6.93) |  |
| Sales and customer service | 1.39 (1.01, 1.92) | 2.38 (1.57, 3.59) | 1.45 (0.60, 3.45) | 1.67 (0.69, 3.96) |  |
| Plant and machine operatives | 3.00 (2.40, 3.74) | 1.41 (0.79, 2.51) | 2.32 (1.16, 4.58) | 2.00 (0.90, 4.40) |  |
| Elementary trades and labourers | 1.93 (1.47, 2.53) | 2.60 (1.72, 3.91) | 1.45 (0.60, 3.45) | 1.33 (0.50, 3.52) |  |
|  |  |  |  |  |  |
| **Sex** |  |  |  |  |  |
| Male | 52.64 (50.67, 54.61) | 53.35 (49.89, 56.78) | 52.75 (47.45, 58.00) | 55.67 (49.96, 61.22) | 0.789 |
| Female | 47.36 (45.39, 49.33) | 46.65 (43.22, 50.11) | 47.25 (42.00, 52.55) | 44.33 (38.78, 50.04) |  |
|  |  |  |  |  |  |
| **Working hours** |  |  |  |  |  |
| Mean (hours/week) | 37.35 (36.91, 37.80) | 36.05 (35.30, 36.79) | 37.20 (36.03, 38.38) | 36.38 (35.04, 37.71) | 0.021 |
|  |  |  |  |  |  |
| **Lifestyle factors** |  |  |  |  |  |
| **Alcohol consumption status** |  |  |  |  |  |
| Current drinker | 95.86 (95.02, 96.56) | 95.56 (93.86, 96.81) | 95.94 (93.25, 97.59) | 97.00 (94.32, 98.44) | 0.691 |
| Non-drinker | 4.14 (3.44, 4.98) | 4.44 (3.19, 6.14) | 4.06 (2.41, 6.75) | 3.00 (1.56, 5.68) |  |
|  |  |  |  |  |  |
| **Body mass index** |  |  |  |  |  |
| Mean (kg/m^2^) | 26.85 (26.67, 27.02) | 25.55 (25.30, 25.81) | 26.38 (25.90, 26.87) | 26.85 (26.35, 27.36) | <0.001 |
|  |  |  |  |  |  |
| **Heavy manual/physical work** |  |  |  |  |  |
| Always/usually | 8.11 (7.13, 9.21) | 4.11 (3.01, 5.60) | 5.80 (3.76, 8.83) | 6.67 (4.33, 10.13) | <0.001 |
| Sometimes/rarely/never | 91.89 (90.79, 92.87) | 95.89 (94.40, 96.99) | 94.20 (91.17, 96.24) | 93.33 (89.87, 95.67) |  |
|  |  |  |  |  |  |
| **Mainly walking or standing at work** |  |  |  |  |  |
| Always/usually | 25.64 (24.00, 27.36) | 20.13 (17.55, 22.98) | 21.45 (17.42, 26.12) | 20.67 (16.44, 25.65) | 0.003 |
| Sometimes/rarely/never | 74.36 (72.64, 76.00) | 79.87 (77.02, 82.45) | 78.55 (73.88, 82.58) | 79.33 (74.35, 83.56) |  |
|  |  |  |  |  |  |
| **Non-commuting mode of transport** |  |  |  |  |  |
| Active | 39.21 (37.39, 41.07) | 79.33 (76.55, 81.86) | 48.70 (43.43, 53.99) | 60.00 (54.32, 65.43) | <0.001 |
| Inactive | 60.79 (58.93, 62.61) | 20.67 (18.14, 23.45) | 51.30 (46.01, 56.57) | 40.00 (34.57, 45.68) |  |
|  |  |  |  |  |  |
| **Smoking status** |  |  |  |  |  |
| Current smoker | 5.75 (4.93, 6.70) | 4.98 (3.72, 6.63) | 6.38 (4.23, 9.51) | 7.33 (4.87, 10.91) | 0.480 |
| Non-smoker | 94.25 (93.30, 95.07) | 95.02 (93.37, 96.28) | 93.62 (90.49, 95.77) | 92.67 (89.09, 95.13) |  |
|  |  |  |  |  |  |
| **Vigorous physical activity** |  |  |  |  |  |
| Mean (minutes/week) | 27.77 (26.39, 29.16) | 32.90 (30.54, 35.27) | 25.57 (22.71, 28.44) | 34.17 (29.55, 38.79) | <0.001 |
|  |  |  |  |  |  |
| **Walking for pleasure** |  |  |  |  |  |
| Mean (minutes/week) | 68.21 (63.62, 72.79) | 74.04 (65.42, 82.66) | 83.00 (70.44, 95.56) | 72.42 (59.44, 85.41) | 0.139 |
|  |  |  |  |  |  |
| **Health factors** |  |  |  |  |  |
| **Bereavement in the preceding two years** |  |  |  |  |  |
| Yes | 21.29 (19.81, 22.84) | 18.83 (16.43, 21.49) | 15.94 (12.43, 20.22) | 21.00 (16.74, 26.01) | 0.050 |
| No | 78.71 (77.16, 80.19) | 81.17 (78.51, 83.57) | 84.06 (79.78, 87.57) | 79.00 (73.99, 83.26) |  |
|  |  |  |  |  |  |
| **Bone fracture in the preceding five years** |  |  |  |  |  |
| Yes | 7.29 (6.36, 8.33) | 7.58 (5.98, 9.56) | 8.12 (5.65, 11.52) | 5.33 (3.28, 8.55) | 0.449 |
| No | 92.71 (91.67, 93.64) | 92.42 (90.44, 94.02) | 91.88 (88.48, 94.35) | 94.67 (91.45, 96.72) |  |
|  |  |  |  |  |  |
| **Financial difficulty in the preceding two years** |  |  |  |  |  |
| Yes | 8.71 (7.71, 9.83) | 7.14 (5.63, 9.02) | 7.54 (5.17, 10.86) | 9.33 (6.51, 13.21) | 0.381 |
| No | 91.29 (90.17, 92.29) | 92.86 (90.98, 94.37) | 92.46 (89.14, 94.83) | 90.67 (86.79, 93.49) |  |
|  |  |  |  |  |  |
| **Non-vascular condition or disability^b^** |  |  |  |  |  |
| Yes | 17.39 (15.98, 18.90) | 16.99 (14.65, 19.62) | 18.84 (15.04, 23.34) | 18.33 (14.33, 23.15) | 0.871 |
| No | 82.61 (81.10, 84.02) | 83.01 (80.38, 85.35) | 81.16 (76.66, 84.96) | 81.67 (76.85, 85.67) |  |
|  |  |  |  |  |  |
| **Serious illness or injury in the preceding two years^c^** |  |  |  |  |  |
| Yes | 17.54 (16.16, 19.00) | 20.78 (18.24, 23.57) | 18.55 (14.78, 23.03) | 19.67 (15.53, 24.58) | 0.189 |
| No | 82.46 (81.00, 83.84) | 79.22 (76.43, 81.76) | 81.45 (76.97, 85.22) | 80.33 (75.42, 84.47) |  |
|  |  |  |  |  |  |
| **Vascular condition^d^** |  |  |  |  |  |
| Yes | 17.71 (16.28, 19.24) | 13.20 (11.09, 15.65) | 9.86 (7.11, 13.50) | 13.67 (10.21, 18.07) | <0.001 |
| No | 82.29 (80.76, 83.72) | 86.80 (84.35, 88.91) | 90.14 (86.50, 92.89) | 86.33 (81.93, 89.79) |  |
| *^a^Inactive commuting defined as any commute by 'car or motor vehicle' only, with active commuting defined as using any other mode or combination of modes.* | | | | | |
| *^b^Defined according to whether participants reported ever receiving a doctor’s diagnosis for diabetes, cancer or 'any other serious medical conditions or disabilities'.* | | | | | |
| *^c^Defined as any self-reported 'serious illness or injury' to the participant or a close relative in the two years preceding baseline.* | | | | | |
| *^d^Defined according to whether participants reported ever receiving a doctor’s diagnosis for angina, heart attack, high blood pressure or stroke.* | | | | | |
| *N refers to the total number of observed transitions. Differences in covariate means and proportions by baseline commute mode were tested by way of a Wald test.* | | | | | |

**Appendix 2b Detailed breakdown of baseline characteristics for each of the four commute transition categories among commuters who were symptomatic at baseline within the analytical sample**

|  | **Symptomatic at baseline** | | | | |
| --- | --- | --- | --- | --- | --- |
|  | **Stable inactive (N=939)** | **Stable active (N=296)** | **Inactive to active (N=139)** | **Active to inactive (N=112)** | **Difference** |
| **Baseline covariates** | **% (95% CI)** | **% (95% CI)** | **% (95% CI)** | **% (95% CI)** | **p-value** |
| **Commute characteristics** |  |  |  |  |  |
| **Commute distance** |  |  |  |  |  |
| Mean (miles) | 12.16 (11.19, 13.13) | 10.57 (7.28, 13.86) | 13.09 (9.88, 16.30) | 10.40 (5.74, 15.06) | 0.627 |
|  |  |  |  |  |  |
| **Commute frequency** |  |  |  |  |  |
| Mean (trips/week) | 4.64 (4.57, 4.72) | 4.52 (4.39, 4.65) | 4.75 (4.34, 5.16) | 4.57 (4.36, 4.78) | 0.345 |
|  |  |  |  |  |  |
| **Severity of depressive symptoms** |  |  |  |  |  |
| **Depression score** |  |  |  |  |  |
| Mean | 1.84 (1.77, 1.91) | 1.66 (1.58, 1.81) | 1.68 (1.51, 1.84) | 1.68 (1.50, 1.86) | 0.045 |
|  |  |  |  |  |  |
| **Socio-demographic and occupational factors** |  |  |  |  |  |
| **Age** |  |  |  |  |  |
| Mean (years) | 50.24 (49.85, 50.64) | 49.69 (49.06, 50.32) | 49.72 (48.69, 50.74) | 48.95 (47.80, 50.10) | 0.108 |
|  |  |  |  |  |  |
| **Ethnicity** |  |  |  |  |  |
| White | 95.42 (93.88, 96.59) | 94.59 (91.33, 96.68) | 98.56 (94.34, 99.65) | 97.32 (91.89, 99.15) | 0.037 |
| Non-white | 4.58 (3.41, 6.12) | 5.41 (3.32, 8.67) | 1.44 (0.35, 5.66) | 2.68 (0.85, 8.11) |  |
|  |  |  |  |  |  |
| **Gross household income** |  |  |  |  |  |
| <£18,000 | 3.41 (2.42, 4.78) | 5.74 (3.59, 9.07) | 2.16 (0.69, 6.56) | 7.14 (3.57, 13.77) | 0.604 |
| £18,000-30,999 | 18.85 (16.42, 21.55) | 19.26 (15.07, 24.27) | 15.83 (10.60, 22.97) | 18.75 (12.47, 27.20) |  |
| £31,000-51,999 | 33.33 (30.38, 36.43) | 34.46 (29.13, 40.21) | 36.69 (29.01, 45.11) | 34.82 (26.47, 44.22) |  |
| £52,000-100,000 | 37.81 (34.68, 41.03) | 34.46 (29.09, 40.26) | 40.29 (32.37, 48.74) | 31.25 (23.25, 40.55) |  |
| >£100,000 | 6.60 (5.14, 8.44) | 6.08 (3.86, 9.46) | 5.04 (2.40, 10.27) | 8.04 (4.19, 14.86) |  |
|  |  |  |  |  |  |
| **Highest educational qualification** |  |  |  |  |  |
| University or college degree | 42.81 (39.58, 46.11) | 58.45 (52.56, 64.10) | 48.92 (40.62, 57.29) | 53.57 (44.18, 62.72) | 0.001 |
| Further education | 15.34 (13.12, 17.85) | 13.51 (10.05, 17.93) | 15.83 (10.60, 22.97) | 12.50 (7.48, 20.15) |  |
| Higher secondary education | 21.19 (18.62, 24.01) | 12.50 (9.17, 16.81) | 17.99 (12.39, 25.37) | 22.32 (15.46, 31.10) |  |
| Secondary education | 8.09 (6.44, 10.12) | 5.41 (3.32, 8.67) | 8.63 (4.93, 14.68) | 2.68 (0.85, 8.11) |  |
| Vocational qualifications | 6.50 (5.06, 8.30) | 3.38 (1.82, 6.18) | 4.32 (1.93, 9.36) | 5.36 (2.40, 11.54) |  |
| Other professional qualifications | 3.19 (2.24, 4.53) | 3.04 (1.58, 5.76) | 2.88 (1.07, 7.50) | 1.79 (0.44, 7.00) |  |
| None of the above | 2.88 (1.93, 4.27) | 3.72 (2.07, 6.60) | 1.44 (0.35, 5.66) | 1.79 (0.44, 7.00) |  |
|  |  |  |  |  |  |
| **Marital status** |  |  |  |  |  |
| Married or partnered | 86.05 (83.56, 88.22) | 88.85 (84.69, 91.99) | 89.21 (82.77, 93.43) | 83.93 (75.78, 89.71) | 0.376 |
| Not married or partnered | 13.95 (11.78, 16.44) | 11.15 (8.01, 15.31) | 10.79 (6.57, 17.23) | 16.07 (10.29, 24.22) |  |
|  |  |  |  |  |  |
| **Occupational grade** |  |  |  |  |  |
| Managerial | 21.62 (19.08, 24.39) | 13.85 (10.28, 18.41) | 20.14 (14.22, 27.73) | 16.96 (11.01, 25.22) | 0.166 |
| Professional | 28.54 (25.68, 31.59) | 29.05 (24.01, 34.68) | 34.53 (27.02, 42.91) | 27.68 (20.09, 36.82) |  |
| Associate professional | 16.72 (14.46, 19.25) | 17.23 (13.24, 22.11) | 17.27 (11.79, 24.57) | 14.29 (8.87, 22.20) |  |
| Administrative and secretarial | 14.80 (12.60, 17.32) | 20.27 (15.97, 25.37) | 13.67 (8.84, 20.54) | 24.11 (16.99, 33.02) |  |
| Skilled trades | 5.22 (3.96, 6.84) | 5.07 (3.07, 8.25) | 5.76 (2.88, 11.17) | 5.36 (2.40, 11.54) |  |
| Professional services | 4.69 (3.48, 6.28) | 6.42 (4.12, 9.86) | 5.04 (2.40, 10.27) | 4.46 (1.84, 10.41) |  |
| Sales and customer service | 1.60 (0.93, 2.72) | 2.03 (0.91, 4.46) | 1.44 (0.35, 5.66) | 2.68 (0.85, 8.11) |  |
| Plant and machine operatives | 4.15 (3.03, 5.68) | 3.04 (1.48, 6.15) | 1.44 (0.35, 5.66) | 1.79 (0.44, 7.00) |  |
| Elementary trades and labourers | 2.66 (1.75, 4.03) | 3.04 (1.58, 5.76) | 0.72 (0.10, 5.04) | 2.68 (0.85, 8.11) |  |
|  |  |  |  |  |  |
| **Sex** |  |  |  |  |  |
| Male | 47.82 (44.52, 51.14) | 46.62 (40.80, 52.53) | 46.04 (37.84, 54.47) | 42.86 (33.90, 52.31) | 0.779 |
| Female | 52.18 (48.86, 55.48) | 53.38 (47.47, 59.20) | 53.96 (45.53, 62.16) | 57.14 (47.69, 66.10) |  |
|  |  |  |  |  |  |
| **Working hours** |  |  |  |  |  |
| Mean (hours/week) | 37.25 (36.50, 38.00) | 35.49 (34.35, 36.64) | 38.55 (36.83, 40.28) | 36.96 (34.12, 37.80) | 0.011 |
|  |  |  |  |  |  |
| **Lifestyle factors** |  |  |  |  |  |
| **Alcohol consumption status** |  |  |  |  |  |
| Current drinker | 95.10 (93.52, 96.31) | 93.92 (90.52, 96.15) | 95.68 (90.64, 98.07) | 91.07 (84.06, 95.18) | 0.436 |
| Non-drinker | 4.90 (3.69, 6.48) | 6.08 (3.85, 9.48) | 4.32 (1.93, 9.36) | 8.93 (4.82, 15.94) |  |
|  |  |  |  |  |  |
| **Body mass index** |  |  |  |  |  |
| Mean (kg/m^2^) | 27.21 (26.89, 27.53) | 25.83 (25.37, 26.30) | 27.10 (26.36, 27.83) | 26.97 (26.08, 27.85) | <0.001 |
|  |  |  |  |  |  |
| **Heavy manual/physical work** |  |  |  |  |  |
| Always/usually | 9.58 (7.78, 11.76) | 9.12 (6.24, 13.15) | 7.91 (4.40, 13.82) | 8.04 (4.19, 14.86) | 0.882 |
| Sometimes/rarely/never | 90.42 (88.24, 92.22) | 90.88 (86.85, 93.76) | 92.09 (86.18, 95.60) | 91.96 (85.14, 95.81) |  |
|  |  |  |  |  |  |
| **Mainly walking or standing at work** |  |  |  |  |  |
| Always/usually | 27.90 (25.04, 30.95) | 24.66 (19.96, 30.06) | 27.34 (20.50, 35.44) | 29.46 (21.66, 38.69) | 0.693 |
| Sometimes/rarely/never | 72.10 (69.05, 74.96) | 75.34 (69.94, 80.04) | 72.66 (64.56, 79.50) | 70.54 (61.31, 78.34) |  |
|  |  |  |  |  |  |
| **Non-commuting mode of transport** |  |  |  |  |  |
| Active | 38.02 (34.91, 41.23) | 80.07 (75.10, 84.25) | 47.48 (39.22, 55.88) | 60.71 (51.25, 69.43) | <0.001 |
| Inactive | 61.98 (58.77, 65.09) | 19.93 (15.75, 24.90) | 52.52 (44.12, 60.78) | 39.29 (30.57, 48.75) |  |
|  |  |  |  |  |  |
| **Smoking status** |  |  |  |  |  |
| Current smoker | 8.41 (6.76, 10.42) | 8.11 (5.39, 12.01) | 5.04 (2.40, 10.27) | 10.71 (6.13, 18.06) | 0.320 |
| Non-smoker | 91.59 (89.58, 93.24) | 91.89 (87.99, 94.61) | 94.96 (89.73, 97.60) | 89.29 (81.94, 93.87) |  |
|  |  |  |  |  |  |
| **Vigorous physical activity** |  |  |  |  |  |
| Mean (minutes/week) | 23.37 (21.24, 25.50) | 28.00 (24.33, 31.67) | 26.00 (18.29, 33.71) | 27.99 (21.06, 34.92) | 0.141 |
|  |  |  |  |  |  |
| **Walking for pleasure** |  |  |  |  |  |
| Mean (minutes/week) | 53.90 (48.03, 59.76) | 67.60 (53.55, 81.65) | 75.79 (59.16, 92.42) | 82.11 (60.45, 103.77) | 0.004 |
|  |  |  |  |  |  |
| **Health factors** |  |  |  |  |  |
| **Bereavement in the preceding two years** |  |  |  |  |  |
| Yes | 21.09 (18.59, 23.82) | 20.27 (16.02, 25.31) | 30.94 (23.73, 39.20) | 23.21 (16.22, 32.07) | 0.108 |
| No | 78.91 (76.18, 81.41) | 79.73 (74.69, 83.98) | 69.06 (60.80, 76.27) | 76.79 (67.93, 83.78) |  |
|  |  |  |  |  |  |
| **Bone fracture in the preceding five years** |  |  |  |  |  |
| Yes | 5.43 (4.15, 7.08) | 8.78 (6.04, 12.61) | 8.63 (4.93, 14.68) | 12.50 (7.48, 20.15) | 0.038 |
| No | 94.57 (92.92, 95.85) | 91.22 (87.39, 93.96) | 91.37 (85.32, 95.07) | 87.50 (79.85, 92.52) |  |
|  |  |  |  |  |  |
| **Financial difficulty in the preceding two years** |  |  |  |  |  |
| Yes | 21.62 (19.06, 24.42) | 20.95 (16.66, 25.99) | 18.71 (13.00, 26.16) | 20.54 (13.96, 29.16) | 0.875 |
| No | 78.38 (75.58, 80.94) | 79.05 (74.01, 83.34) | 81.29 (73.84, 87.00) | 79.46 (70.84, 86.04) |  |
|  |  |  |  |  |  |
| **Non-vascular condition or disability^b^** |  |  |  |  |  |
| Yes | 21.09 (18.56, 23.85) | 19.93 (15.72, 24.95) | 23.02 (16.70, 30.85) | 22.32 (15.46, 31.10) | 0.890 |
| No | 78.91 (76.15, 81.44) | 80.07 (75.05, 84.28) | 76.98 (69.15, 83.30) | 77.68 (68.90, 84.54) |  |
|  |  |  |  |  |  |
| **Serious illness or injury in the preceding two years^c^** |  |  |  |  |  |
| Yes | 20.66 (18.18, 23.39) | 23.65 (19.04, 28.97) | 25.90 (19.23, 33.92) | 26.79 (19.31, 35.87) | 0.282 |
| No | 79.34 (76.61, 81.82) | 76.35 (71.03, 80.96) | 74.10 (66.08, 80.77) | 73.21 (64.13, 80.69) |  |
|  |  |  |  |  |  |
| **Vascular condition^d^** |  |  |  |  |  |
| Yes | 16.61 (14.28, 19.24) | 18.24 (14.15, 23.20) | 17.27 (11.79, 24.57) | 14.29 (8.87, 22.20) | 0.792 |
| No | 83.39 (80.76, 85.72) | 81.76 (76.80, 85.85) | 82.73 (75.43, 88.21) | 85.71 (77.80, 91.13) |  |
| *^a^Inactive commuting defined as any commute by 'car or motor vehicle' only, with active commuting defined as using any other mode or combination of modes.* | | | | | |
| *^b^Defined according to whether participants reported ever receiving a doctor’s diagnosis for diabetes, cancer or 'any other serious medical conditions or disabilities'.* | | | | | |
| *^c^Defined as any self-reported 'serious illness or injury' to the participant or a close relative in the two years preceding baseline.* | | | | | |
| *^d^Defined according to whether participants reported ever receiving a doctor’s diagnosis for angina, heart attack, high blood pressure or stroke.* | | | | | |
| *N refers to the total number of observed transitions. Differences in covariate means and proportions by baseline commute mode were tested by way of a Wald test.* | | | | | |

**Appendix 3 Mean severity of depressive symptoms at baseline and follow-up by commute mode**

|  | **Severity of depressive symptoms** | |  |  |
| --- | --- | --- | --- | --- |
|  | **Baseline** | **Follow-up** |  |  |
| **Changes in travel mode over time** | **Mean (SD)** | **Mean (SD)** | **Change score** | **P for difference in change scores** |
| **Asymptomatic** |  |  |  |  |
| Stable inactive (N=2,800) | 0.0 (0.00) | 0.23 (0.64) | 0.23 | 0.130 |
| Stable active (N=924) | 0.0 (0.00) | 0.18 (0.55) | 0.18 |  |
| Inactive to active (N=345) | 0.0 (0.00) | 0.23 (0.65) | 0.23 |  |
| Active to inactive (N=300) | 0.0 (0.00) | 0.21 (0.61) | 0.21 |  |
|  |  |  |  |  |
| **Symptomatic** |  |  |  |  |
| Stable inactive (N=939) | 1.84 (1.05) | 1.06 (1.30) | -0.79 | 0.876 |
| Stable active (N=296) | 1.70 (0.94) | 0.88 (1.19) | -0.82 |  |
| Inactive to active (N=139) | 1.68 (0.97) | 0.91 (1.23) | -0.77 |  |
| Active to inactive (N=112) | 1.68 (0.97) | 0.97 (1.35) | -0.71 |  |
|  |  |  |  |  |

**Appendix 4 Detailed breakdown of commute mode at baseline and follow-up within the analytical sample**

|  |  |  |  |  |  |  |
| --- | --- | --- | --- | --- | --- | --- |
|  | **Follow-up modal category** | | | | | |
|  | **Car only** | **Car and active transport** | **Car and public and active transport** | **Public and active transport** | **Walking only** | **Cycling or walking and cycling** |
| **Baseline modal category** | **% (n)** | **% (n)** | **% (n)** | **% (n)** | **% (n)** | **% (n)** |
| **Asymptomatic at baseline** |  |  |  |  |  |  |
| Car only | **64.09 (2,800)** | 3.04 (133) | 2.88 (126) | 1.05 (46) | 0.69 (30) | 0.23 (10) |
| Car and active transport | 2.61 (114) | **4.17 (182)** | 0.53 (23) | 0.18 (8) | 0.46 (20) | 0.32 (14) |
| Car and public and active transport | 3.00 (131) | 0.53 (23) | **3.87 (169)** | 0.96 (42) | 0.05 (2) | 0.18 (8) |
| Public and active transport | 0.60 (26) | 0.11 (5) | 0.69 (30) | **3.71 (162)** | 0.23 (10) | 0.34 (15) |
| Walking only | 0.48 (21) | 0.25 (11) | 0.07 (3) | 0.16 (7) | **2.20 (96)** | 0.07 (3) |
| Cycling or walking and cycling | 0.18 (8) | 0.16 (7) | 0.14 (6) | 0.14 (6) | 0.07 (3) | **1.58 (69)** |
|  |  |  |  |  |  |  |
| **Symptomatic at baseline** |  |  |  |  |  |  |
| Car only | **63.19 (939)** | 3.16 (47) | 3.16 (47) | 2.09 (31) | 0.61 (9) | 0.34 (5) |
| Car and active transport | 3.16 (47) | **3.36 (50)** | 0.54 (8) | 0.13 (2) | 0.13 (2) | 0.34 (5) |
| Car and public and active transport | 3.50 (52) | 0.34 (5) | **3.50 (52)** | 1.21 (18) | 0.13 (2) | 0.00 (0) |
| Public and active transport | 0.47 (7) | 0.20 (3) | 0.74 (11) | **4.17 (62)** | 0.47 (7) | 0.20 (3) |
| Walking only | 0.34 (5) | 0.20 (3) | 0.20 (3) | 0.13 (2) | **1.75 (26)** | 0.07 (1) |
| Cycling or walking and cycling | 0.07 (1) | 0.20 (3) | 0.13 (2) | 0.00 (0) | 0.13 (2) | **1.62 (24)** |
| *'Active transport' is defined as walking, cycling or walking and cycling.*  *n refers to the total number of transitions; some participants will be counted twice.* | | | | | | |

1. Ferrari AJ, Charlson FJ, Norman RE, Patten SB, Freedman G, Murray CJ, et al. Burden of depressive disorders by country, sex, age, and year: findings from the global burden of disease study 2010. PLoS Med. 2013;10(11):e1001547. [↑](#endnote-ref-1)
2. Office for National Statistics. Standard Occupational Classification 2000. Volume 1: Structure and descriptions of unit groups. London: The Stationery Office; 2000. [↑](#endnote-ref-2)
3. American Psychiatric Association. Chapter 5: Depressive Disorder. In Diagnostic and Statistical Manual of Mental Disorders (Fifth ed.). Arlington, VA: American Psychiatric Publishing; 2013. [↑](#endnote-ref-3)
4. Maj M. “Clinical judgment” and the DSM-5 diagnosis of major depression. World Psychiatry. 2013; 12(2): 89–91. [↑](#endnote-ref-4)
